# Pandemic: Psychiatric-Mental Health Nurses Providing Hope in the Midst of Chaos

**DOI:** 10.1177/1078390320918547

**Published:** 2020-05-04

**Authors:** Sattaria “Tari” Dilks

**Affiliations:** 1 Sattaria “Tari” Dilks, DNP, APRN, PMHNP-BC, FAANP, American Psychiatric Nurses Association, Falls Church, VA, USA

Over my past several columns, we have followed a nurse named Janice through her early career. We are going to pause that story and use this column instead to offer support during the current public health emergency. We are with you.

This year has been designated the Year of the Nurse in recognition of our importance to health and well-being. Nurses make up the bulk of the health care workforce and are natural problem solvers and innovators. We therefore stand out as indispensable at any time, but especially during a time of emergency like the current coronavirus pandemic. In 2008, the American Nurses Association (ANA) released a paper, *Adapting Standards of Care Under Extreme Conditions*. It provides vital information for nurses preparing to provide care during unusual and extreme circumstances. It emphasizes that we must stand ready to provide competent care and make appropriate health care decisions in response to extreme circumstances. Every organization has a responsibility to have a plan in place for an emergency response. Similarly, the International Council of Nursing (ICN) offers core competencies in disaster nursing organized into eight domains (ICN, 2019a): preparation and planning, communication, incident management systems safety and security, assessment, intervention, recovery, and law and ethics. I encourage you to explore both resources—personal and professional preparation is key (Grochtdreis et al., 2016) for each of us, as is seeking information from the most reputable sources (as noted in our ethical standards).

In times of global pandemic, uncertainties become the norm. Essential services may be eroded, resources become scarce, individuals’ socioeconomic situations shift, and countless other circumstances are in flux. Effective nursing practice during a time like this requires a utilitarian approach (ICN, 2019a): “What will do the greatest amount of good for the most amount of people?” Congruent with Maslow’s Hierarchy of Needs (Maslow & Lewis, 1987), the essentials for physical survival and safety will take precedence. Some things do not change: “No emergency changes the basic standards of practice, code of ethics, competence, or values of the professional” (ANA, 2008, p. 6).

How does this approach affect those of us in psychiatric-mental health? Though we are not triaging in acute care settings or emergency rooms, we are unquestionably providing essential care and psychosocial support to those affected during this crisis. The ICN (2019b) calls for “the inclusion of mental health and psychosocial support for responders and survivors, and for their families, as part of the health response to disaster and disaster recovery,” (p. 3). This succinctly describes our role. Psychiatric-mental health nurses are critically needed in all disaster responses. Almost everyone is at risk to experience psychological distress and “people with severe mental disorders are especially vulnerable” (World Health Organization [WHO], 2019, p. 1). The Centers for Disease Control and Prevention (CDC; 2020) maintains that persons with preexisting mental health conditions need to continue treatment and be alert to new and worsening symptoms during a public health emergency. The WHO (2019) concurs, emphasizing that access to basic needs and clinical care is a necessity for those with severe mental health needs.

The WHO (2019) has excellent guidelines around mental health in emergencies, recommending that services to meet specific and urgent mental health care needs be available immediately as a part of any health response. Looking at key lessons learned from a psychiatric inpatient unit during the H1N1 pandemic (2010), we know that having in place plans for managing infectious diseases is important, along with an understanding of your community’s unique resources and needs and creatively sharing knowledge and support around mental health. Beyond that, it is our duty to ensure safety and continuity of care for our patients. This means that we must strive to address challenges in our practice environment, such as facilitating access to urgent mental health care. This occurs while navigating clinic closures, discharging patients, utilizing safety measures to prevent the spread of infection, identifying role confusion, and managing our own stress. As always, relying only on information from trusted sources is a necessity.

Unplanned barriers to providing mental health care as we are accustomed under normal circumstances, such as the inability to meet face-to-face, must be overcome. As Miller (2020) points out, measures such as patient isolation and quarantine to prevent the spread of infection are impediments to the treatment of psychiatric disorders on the inpatient unit; this is where essential treatment activities get patients out of their rooms and into a therapeutic milieu. Educate yourself on the reactions to stress you may see in populations affected—worry, worsening chronic health problems, a change in sleep or eating patterns, difficulty sleeping or concentrating, and an increase in the use of substances are a few (CDC, 2020).

During a time of public health crisis, emergency changes to regulations may be implemented to help people obtain the care they need. This includes facilitating our ability to provide care remotely. As pointed out by the ANA (2008), while our scope of practice and ethics do not change in an emergency, “legal structures for health professionals *MAY* change” (p. 6). For example, to ease access to care during the current pandemic, the Office for Civil Rights at the U.S. Department of Health and Human Services (2020) has waived potential penalties for HIPAA (Health Insurance Portability and Accountability Act) violations against providers serving patients through everyday technologies such as Facetime or Skype. As psychiatric nurses we must be able to respond in a nimble manner and provide services remotely as necessary. Becoming knowledgeable in telehealth technologies that are accessible to patients in their homes, as well as the ability to quickly implement those technologies is critical. Telehealth is a viable option for many providers, so long as the right technologies are in place. According to a 2018 *JAPNA* article by Adams et al., telemental health has been shown to have comparable (or even sometimes better) outcomes when compared to in-person care. For a great overview of considerations when implementing telemental health, I encourage you to access this article.

Another source of current information and real-world solutions is our online community. A quick scan of the American Psychiatric Nurses Association’s Member Bridge shows that colleagues everywhere are sharing ways to find balance between necessary safety measures and the well-being of their patients across settings. Conversations cover everything from visitation policies on a child and adolescent inpatient units to helping those in the community dependent on provider-administered injections (while keeping staff safe), to best practice when using telepsychiatry.

Please remember that arming yourself with all this information is pointless if you are too stressed and burnt out to provide care. In the face of a crisis, self-care becomes more crucial than ever. Secondary traumatic stress, also known as comfort fatigue, is real. As health care providers we are a group especially vulnerable (CDC, 2020). I encourage you to appreciate that you will have reactions to the increased stressors you will likely encounter, such as lack of adequate rest, erratic eating schedules, information overload, increased work and personal responsibilities, and limited resources. Check in with yourself to see if you are experiencing any symptoms of secondary traumatic stress. Common symptoms include lowered concentration, rigid thinking, guilt, numbness, sleep disturbance, withdrawal from others, increased heart rate, and muscle and joint pain (U.S. Department of Health and Human Services Administration for Children & Families, n.d.). Recognize what you can and cannot control to prioritize your health: eat healthily, drink plenty of fluids, and try to make time for relaxation regimes and pleasurable activities. Give your mind a rest from the constant flow of information—the CDC (2020) recommends taking breaks from media coverage of COVID-19. Wei (2018) offers these strategies for caregivers to activate their parasympathetic nervous systems to combat stress: exercise self-compassion, practice breath awareness for 10 minutes, try a mind–body practice such as yoga, eat regularly scheduled meals, and avoid foods that increase inflammation in the body. Also, use a nighttime routine to promote quality sleep and maintain your social connections. It is easy to play up the importance of self-care to our patients while downplaying it to ourselves. Resist that urge.

Looking to the future, we need to be ready to respond to the new mental health needs that will emerge after people have lived through a disaster or crisis. Recovery from this pandemic will depend much on us and the mental health system. We know that reactions to the event should be anticipated as part of recovery (Hughes, 2010). The impact of isolation and fear should be assessed. Those with less resilience will likely be in urgent need of care. What support services will they need? What support services do YOU need?

This emergency will lessen, and we will move on to the postevent phase. When that time comes, the ANA (2008, p. 18) recommends the following steps for nurses after a disaster event:

- Participate in postevent evaluation
- Do a psychosocial needs assessment for self and family and seek assistance if indicated
- Participate in activities to facilitate return to preevent status

As a nursing community, it will be important for us to explore how to equip the next generation of nurses to deal with the likely fallout from this pandemic and be prepared for the next. Grochtdreis et al. (2016) emphasizes that response training and preparation for nurses is essential, and offers the following recommendation for education: “Nursing educators should prepare nurses for disasters, by adjusting the curricula and by meeting the increased need for education and training in disaster nursing for all groups of nurses” (p. 2).

As we move forward it is important to take note of the lessons learned and improve our contingency plans for the next time something like this happens. We should ask ourselves:

What went wrong?
What went right?
What do we need to fix?
What do we need to build?

We have the opportunity before us to assess honestly what has happened across the world and make changes for the next time. Let us not waste it.

I hope that each of you finds health and safety during this time and that we all remain committed to the best care possible for our patients, families, each other, and ourselves.
